# Evaluation of a Restoration Algorithm Applied to Clipped Tibial Acceleration Signals

**DOI:** 10.3390/s23104609

**Published:** 2023-05-10

**Authors:** Zoe Y. S. Chan, Chloe Angel, Daniel Thomson, Reed Ferber, Sharon M. H. Tsang, Roy T. H. Cheung

**Affiliations:** 1 Faculty of Kinesiology, University of Calgary, Calgary, AB T2N 1N4, Canada; zoe.chan1@ucalgary.ca (Z.Y.S.C.);; 2 Department of Rehabilitation Sciences, The Hong Kong Polytechnic University, Hong Kong SAR, China; 3 School of Health Sciences, Western Sydney University, Penrith, NSW 2751, Australia

**Keywords:** operating range, accelerometer, peak tibial acceleration, interpolation, wearable sensors

## Abstract

Wireless accelerometers with various operating ranges have been used to measure tibial acceleration. Accelerometers with a low operating range output distorted signals and have been found to result in inaccurate measurements of peaks. A restoration algorithm using spline interpolation has been proposed to restore the distorted signal. This algorithm has been validated for axial peaks within the range of 15.0–15.9 *g*. However, the accuracy of peaks of higher magnitude and the resultant peaks have not been reported. The purpose of the present study is to evaluate the measurement agreement of the restored peaks using a low-range accelerometer (±16 *g*) against peaks sampled using a high-range accelerometer (±200 *g*). The measurement agreement of both the axial and resultant peaks were examined. In total, 24 runners were equipped with 2 tri-axial accelerometers at their tibia and completed an outdoor running assessment. The accelerometer with an operating range of ±200 *g* was used as reference. The results of this study showed an average difference of −1.40 ± 4.52 *g* and −1.23 ± 5.48 *g* for axial and resultant peaks. Based on our findings, the restoration algorithm could skew data and potentially lead to incorrect conclusions if used without caution.

## 1. Introduction

Running is popular around the world. It is among the top five most popular physical exercise practiced by individuals of all ages [1]. For more than 40 years, sport scientists have been quantifying the running gait to assess performance, evaluate footwear and understand running-related injuries. One of the most common tools used in gait analyses is the force plate [2]. Force plates measure three-dimensional ground reaction forces and are usually installed within research labs or embedded on treadmills. However, the equipment size and installation requirements have constrained study protocols to treadmills and/or within lab-settings, which could impact the natural running pattern. In fact, there has been increasing evidence of biomechanical differences between running indoors and running in ‘real-world’ environments [3,4,5].

Segmental accelerations have been used to predict measures of ground reaction forces and loading rates [6,7,8]. Early measurements of segmental acceleration were invasive with the use of bone-mounted accelerometers [9]. In recent years, the use of skin-mounted accelerometers has become popular. Wireless skin-mounted accelerometers provide a non-invasive alternative to valid and reliable measurements of segmental acceleration [10]. One of the most common sites for accelerometer attachment is the shank [10]. Axial and resultant peak tibial accelerations (PTA), registered by skin-mounted accelerometers at the shank, have been shown to be correlated with vertical loading rates [6,11]. Technological advancement in wearable sensors has offered an alternative to force plates, allowing for monitoring and analysis of running mechanics in any environment.

In a recent systematic review on wearables used for running gait analysis, the operating range of accelerometers used was between ±2 *g* (*g* = 9.81 m/s^2^) and ±200 *g*, with ±16 *g* being the most common [10]. However, studies have reported PTA of up to 24.6 *g* during overground running [12], exceeding the operating range of some wireless sensor systems popular among research groups. In the case of the signal range exceeding the sensor operating range, data clipping may occur. A clipped signal is distorted, displaying flat-cut-offs, and the amplitude of the peak is not registered. Mitschke et al. have reported up to 6.25% and 28.17% decrease in accuracy for PTA measurement when comparing an operating range of ±16 *g* and ±8 *g* to ±70 *g*, respectively [13]. A restoration algorithm has been proposed by Ruder et al. for clipped tibial acceleration signals [14] based on spline interpolation to reconstruct the missing peaks. This algorithm has been applied to another study which compared PTA between treadmill and real-world running [15]. While Ruder et al. [14] concluded that the algorithm was sufficient to reconstruct peaks accurately, the validation was limited to a narrow range of peaks from 15.0 to 15.9 *g*; the accuracy of the algorithm beyond 15.9 *g* is unknown. Furthermore, only axial PTA was evaluated in the aforementioned study. The resultant PTA, which is derived from the vector resultant of the tri-axial accelerometer [16,17], has demonstrated higher between-sessions reliability. Signal clipping can occur in any axis, and the restoration algorithm could be applied to each axis separately. A comprehensive evaluation of the restoration algorithm on peaks of higher magnitude and other commonly reported PTA variables is therefore merited.

Hence, this study aims to evaluate the measurement agreement of the restored tibial acceleration signals sampled using a low-range accelerometer (±16 *g*) and acceleration signals sampled using a high-range accelerometer (±200 *g*). The measurement agreement of the axial and resultant PTA derived from the two signals are compared. Based on the findings of the previous validation, we hypothesize underestimation of both axial and resultant PTA using acceleration signals restored by the algorithm.

## 2. Materials and Methods

Previous validation for the restoration algorithm used 18,000 steps [14]. Based on an average stride length of 1.8 m and a track of approximately 1000 m, each participant was estimated to contribute 800 to 1000 steps to the data pool for validation. In total, 24 healthy runners (13 males and 11 females; age: 22.2 ± 4.7 years; height: 1.7 ± 0.1 m; weight: 68.4 ± 12.7 kg) participated in this study. All participants had no history of lower limb surgery and were free from lower limb musculoskeletal injury at the time of data collection. All participants gave their informed consent for inclusion before they participated in the study. The study was conducted in accordance with the Declaration of Helsinki, and the protocol was reviewed and approved by the Human Ethics Committee of Western Sydney University (Project identification code: H14514).

All participants completed a single-session running assessment at the Campbelltown Campus, Western Sydney University. Two dual-accelerometer inertial measurement units (IMUs) (Blue Trident Dual g IMU, Vicon, New Zealand; dimensions: 42 × 27 × 11 mm; weight: 9.5 g) were used in the data collection of this study. Each IMU has two accelerometers encased: Low-G (±16 *g*) and High-G (±200 *g*). Both IMUs were strapped to the anteromedial aspects of both the left and right distal tibiae of the participants [6]. The y-axis of each accelerometer was aligned to the long axis of the tibia. All participants completed a running trial along a 270 m outdoor concrete running track with changes in gradient (−15.8–15.8%). Each participant was told to complete 4 laps at their fastest pace for a total running distance of 1080 m. The accelerometers were set to capture tri-axial acceleration data at 1600 Hz and 1125 Hz for High-G and Low-G, respectively.

Data were processed using custom MATLAB (MathWorks, Inc.; Natick, MA, USA) scripts. A flow diagram of data processing is provided as Supplementary File S1. The Low-G signals were duplicated as Low-G-raw and Low-G-restored, where the latter were further processed using the restoration algorithm. The restoration algorithm was adopted and modified from Ruder et al.’s algorithm [14]. The clipped portion of the acceleration signal which exceeded the operating range of the Low-G accelerator was identified from each of the axes. A window of 3 data points before and after the flat-cutoff was used for the reconstruction of the peak. The spapi() function in 5th order and the fnval() function in MATLAB were used to reconstruct the signal by spline interpolation. The MATLAB script used for restoration is provided as Supplementary File S2. The Low-G-raw, Low-G-restored and High-G acceleration signals were filtered using a 2nd order Butterworth low-pass filter with a cut-off frequency of 85 Hz. Resultant accelerations were computed for each of the three sets of signals as the square root of the sum of the squared acceleration of the x-, y- and z-axes.

The three sets of signals (Low-G-raw, Low-G-restored and High-G) signals were processed with identical scripts to extract axial and resultant PTA. Initial foot contacts were identified as the local minimum that occurred in the 75 ms prior to a local maximum in the resultant acceleration. Within the first 40% of each stride, the maximum value for axial (i.e., y-axis) and resultant PTA were extracted [18]. The axial and resultant PTA for each foot strike were matched with the corresponding peaks obtained from the High-G signal within 200 ms. The peaks obtained from the High-G signal are considered true values. Restored peaks with a value smaller than 16.0 *g* were rejected and removed from the validation.

To facilitate the comparison of our findings to the previous validation study, the axial component of the Low-G-raw signal was artificially clipped at 15.0 *g* by replacing all data points above 15.0 *g* with a value of 15.0 *g*. The same restoration algorithm and axial PTA identification algorithm were applied to the artificial signal. Peaks between 15.0 and 15.9 *g* were selected from the original Low-G-raw signal and were compared to the axial PTA obtained from the restored signal. Same as the previous validation study, restored peaks below 15.0 *g* were rejected and removed from the validation.

A series of two-sample *t*-tests were conducted for each participant to compare the full range of axial and resultant peaks obtained from the Low-G-raw and Low-G-restored signals against the High-G signal. The level of significance was set to an alpha of 0.05. The effect size (Cohen’s *d*) was calculated to quantify the magnitude of difference between the peaks extracted from different signals. The coefficients were interpreted as trivial, small, medium and large effect for *d* < 0.2, 0.2 ≤ *d* < 0.5, 0.5 ≤ *d* < 0.8 and *d* ≥ 0.8, respectively [13,19]. Bland–Altman plots combined with the calculation of 95% confidence limits (limits of agreement; LOA) were used to assess the measurement agreement of axial and resultant PTA derived from the restored signal and the true value. This graphical statistical technique is a generally accepted technique and has been used to assess alternative methods and established methods in biomechanical measurements [20,21].

## 3. Results

No clipping was observed in the High-G signal. Axial and resultant PTA derived from the High-G signals were considered the true value for comparison and validation. A total of 40,855 peaks across 24 participants were obtained. The mean and SD for the axial and resultant PTA were 14.30 ± 6.06 *g* and 20.13 ± 8.97 *g*, respectively.

### 3.1. Two-Sample t-Tests

**Figure 1:**
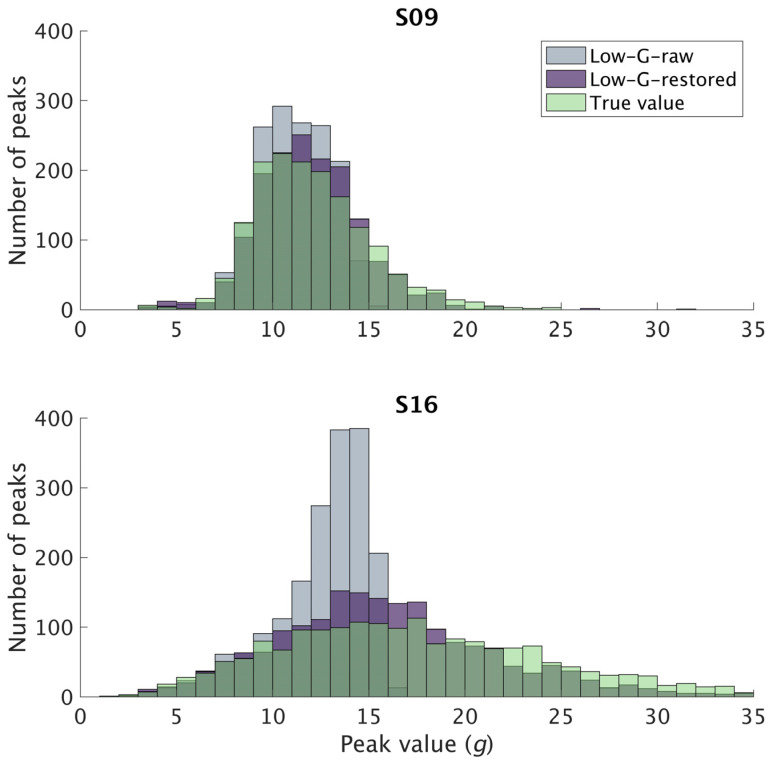
Histogram of axial peaks obtained from the Low-G-raw and Low-G-restored signal and the true value for participant S09 (**top**) and S16 (**bottom**).

Compared against the true values, significant differences (*p* < 0.05) were found in all participants (*n* = 24) for both axial and resultant peaks derived from the Low-G-raw signal. The absolute value of Cohen’s *d* ranged from 0.2 to 1.2 and from 0.2 to 1.6 for axial and resultant PTA, respectively. When using the restored signals, significant differences (*p* < 0.05) were found in 17 participants for axial peaks and in 12 for resultant peaks. The absolute value of Cohen’s *d* ranged from 0.0 to 0.8 and from 0.0 to 0.3 for axial and resultant PTA, respectively. Histograms of axial PTA obtained from the three sets of signals for all participants are provided in Supplementary File S3. Figure 1 shows the distribution of axial PTA in participants S09 and S16. These two participants were selected to demonstrate the effect of the peak magnitude on the result of the *t*-tests. S09 ran with axial PTA of 12.06 ± 3.03 *g*, and a significant but small difference was found between the axial peaks derived from Low-G-raw signal and the true values (*p* < 0.05, *d* = 0.4), but no significant difference was found between the axial peaks derived from Low-G-restored signal and the true values (*p* = 0.17). S16 ran with axial PTA of 17.63 ± 7.51 *g*, and a significant and large difference was found between the axial peaks derived from Low-G-raw signal and the true values (*p* < 0.05, *d* = 0.9). A significant and small difference was also found between the axial peaks derived from Low-G-restored signal and the true values (*p* < 0.05, *d* = 0.3). An inflated number of peaks were also observed around the 13–16 *g* range which could indicate systematic error.

### 3.2. Measurement Agreement of Axial PTA

**Figure 2:**
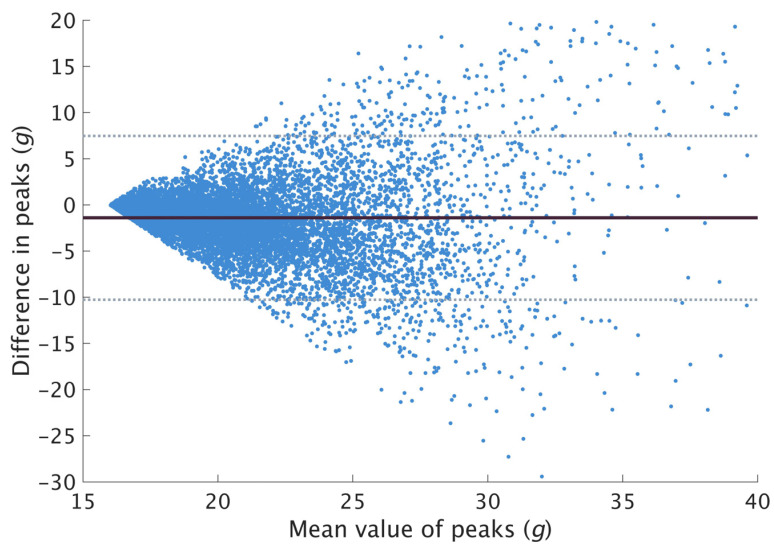
Bland-Altman plot for axial peaks beyond 16 *g*. Solid line represents the mean difference, and the dotted lines represent the LOA (limits of agreement). A negative difference indicates underestimation of restored peaks compared to the true value.

**Figure 3:**
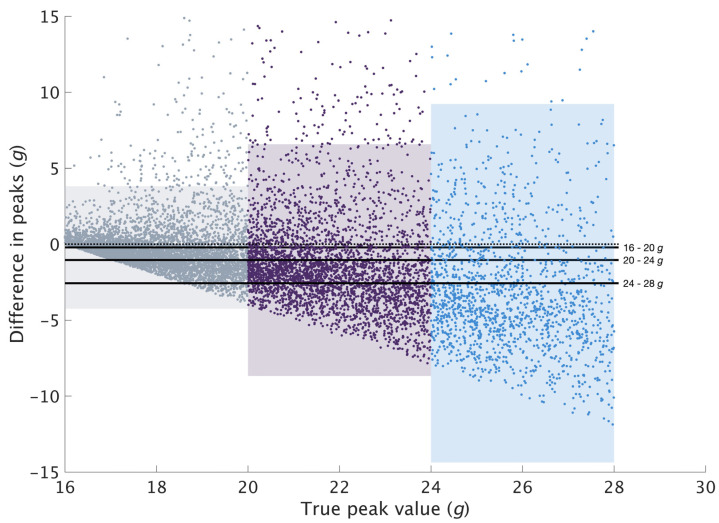
Mean and LOA of the difference in the reconstructed axial peaks against the true value. Labelled solid lines represent the mean difference for each range. The three shaded regions, grey, purple and blue, represent the LOA of the 16–20, 20–24 and 24–28 *g* range, respectively. The dotted line indicates 0 *g*. A negative difference indicates underestimation of restored peaks compared to the true value.

For axial PTA, 13,805 peaks were found to be above 16 *g* based on the true value and 2570 (18.6%) of the reconstructed peaks were rejected for having a value below 16 *g*. A total of 11,235 pairs of peaks were compared and used to examine the measurement agreement for axial PTA. On average, axial PTA were found to be underestimated by 1.40 ± 4.53 *g* (Figure 2). The peaks were then segregated into three groups (16–20 *g*, 20–24 *g* and 24–28 *g*) based on the true value. A total of 5199, 3455 and 1563 pairs of peaks were compared for each range with a mean difference of −0.21 ± 2.06 *g*, −1.04 ± 3.89 *g* and −2.57 ± 6.02 *g*, respectively (Figure 3). The dispersion at higher peak magnitudes of the Bland–Altman plot (Figure 2) and the increasingly wider LOA (Figure 3) suggested weaker agreement at higher peak magnitudes.

### 3.3. Measurement Agreement of Resultant PTA

**Figure 4:**
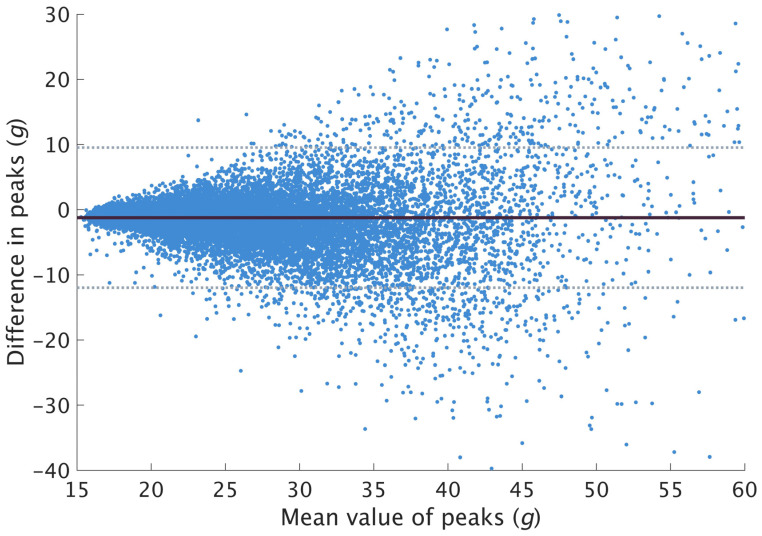
Bland–Altman plot for resultant peaks. Solid line represents the mean difference, and the dotted lines represent the LOA. A negative difference indicates underestimation of restored peaks compared to the true value.

Clipping was observed in each of the three axes. Among all footstrikes across 24 participants, 13,031, 14,388 and 5789 footstrikes exceeded the ±16 *g* range on the x-, y- and *z*-axis, respectively. The algorithm was applied to each of these footstrikes. Each footstrike with at least one of the axes reconstructed was compared and validated against the resultant PTA obtained from the High-G signal. A total of 20,614 pairs of resultant peaks were validated. On average, resultant PTA were found to be underestimated by 1.23 ± 5.48 *g* (Figure 4).

### 3.4. Artificially Clipped Signal (15.0–15.9 g)

**Figure 5:**
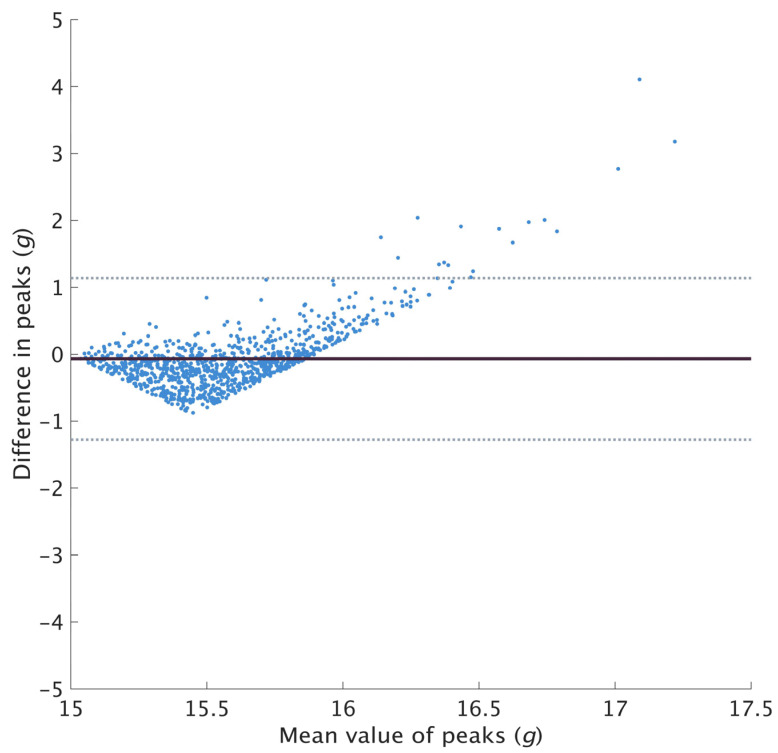
Bland–Altman plot for axial peaks between 15.0 and 15.9 *g*. Solid line represents the mean difference, and the dotted lines represent the LOA. A negative difference indicates underestimation of restored peaks compared to the true value.

A total of 2275 axial peaks were found to be within 15.0–15.9 *g* based on the Low-G-raw signal. Almost half of the reconstructed peaks (45.6%; *n* = 1238) from the artificially clipped signal were rejected by the algorithm for having a value smaller than 15.0 *g*. Among the 1238 pairs of axial PTA compared, the reconstructed peaks were found to be underestimated by 0.03 ± 0.41 *g* (Figure 5).

## 4. Discussion

The aim of the present study is to evaluate the performance of a restoration algorithm on reconstructing peaks from clipped tibial acceleration signals. On average, both the axial and resultant peaks derived from the restored signals were found to be smaller than the true value. The LOA for axial PTA was found to be larger with higher peak magnitudes.

This validation study examined the axial and resultant PTA. These variables are often studied for their potential association with running-related injuries. While the evidence of the relationship remains equivocal, axial and resultant PTA have been frequently used within the literature to examine footwear [22], running surface [18,23,24] and gait retraining [25,26,27]. Compared to previous studies, the axial and resultant PTA values in the current study were on the high side for overground running [16]. Johnson et al. reported average axial and resultant PTA of 11.71 ± 3.66 *g* and 15.72 ± 4.99 *g* at the 12 km checkpoint of a marathon [15], while we found 14.30 ± 6.06 *g* and 20.13 ± 8.97 *g*, respectively. The higher values could be attributed to the running conditions set for this study. This study required a wide range of axial and resultant PTA values which exceed the operating range of the low-range accelerometer (i.e., 16 *g*) for validation of the restoration algorithm. Surface inclination has been found to affect PTA [23,28,29]. The outdoor running route used in this study has a ±15.8% incline/decline, which was selected to facilitate the collection of a wide range of PTA values. Additionally, faster running speeds have previously been found to result in greater axial [6,24] and resultant PTA [6]. Our participants were instructed to run at their fastest pace suitable for a 1000 m sprint to achieve high PTA values. Using this protocol, we were able to collect over 13,000 eligible steps among the 24 participants for validation.

Among the steps used to examine the measurement agreement, the majority of the axial and resultant PTA derived from the restored signal were found to be lower than the true value. Ruder et al. reported similar observations in their validation of axial PTA between 15.0 and 15.9 *g* [14], reporting an average difference of −0.02 ± 0.24 *g*. However, in the current study, the mean difference (−1.40 ± 4.53 *g*) was found to be much larger when comparing peaks beyond 16 *g*. The Bland–Altman plot (Figure 2) showed the data points dispersing and getting wider along the y-axis, with points on the plot exceeding the LOA. This observation indicates a difference in agreement at different peak values. Based on this observation and the results of the previous validation study, it was further hypothesized that the restoration algorithm might be more suitable for restoring peaks with lower magnitudes. Peaks were segregated based on their true values, and we observed increasingly wider LOA in higher peak magnitudes (Figure 3), suggesting weaker agreement. Future studies which expect high peak magnitudes should be mindful of the wide LOA when applying the restoration algorithm.

In biomechanical research, the Bland–Altman plot combined with the calculation of the LOA is a generally accepted technique to evaluate if a new or alternate method agrees sufficiently well with a gold standard [20,21]. However, there are no reference values to determine if the limits are acceptable. The acceptable limit should be set based on clinical or research necessity, with reference to the effect size hypothesized specific to each study [30]. For example, the mean difference in PTA between running on grass and concrete was found to be 0.75 *g* [23]; the restoration algorithm with an LOA between −5.93 and 3.13 *g* may, therefore, not be acceptable. Using the restoration algorithm with a large LOA could result in an error that would mask small differences, leading to invalid interpretations. In this study, we have included the mean difference and LOA for each range for axial PTA and overall resultant PTA. Future studies which intend to use this restoration algorithm should consult the results of this study and exercise caution when interpreting results.

The restoration algorithm used in this study was adopted from the algorithm described in Ruder et al.’s study [14]. However, it was modified for use with the higher sampling frequency in our study and translated from Python to MATLAB. To ensure that our algorithm performed similarly, we artificially removed the peaks between 15.0–15.9 *g* and reconstructed the peaks using the restoration algorithm. Our mean difference was found to be similar to Ruder et al.’s validation study, with a mean difference of less than 0.05 *g*. Both algorithms were also designed to prohibit the value of the reconstructed peak to under 15.0 *g*. In theory, reconstructed peaks should not have a value within the operating range as reconstruction would not have been necessary in such cases. It is unclear how Ruder et al. handled the rejected peaks, and since they did not report the percentage of rejected peaks, a comparison was not possible. In this current study, we rejected 45.6% of the peaks validated between the 15.0 and 15.9 *g* range and 18.6% beyond 16.0 *g*. Rejecting peaks using a set threshold could potentially skew the data; therefore, the percentage of rejected peaks should be reported in future studies that use the restoration algorithm.

A dual-accelerometer device was used in this study, with a ±16 *g* and a ±200 *g* accelerometer encased. This device was selected for the ease to synchronize both High-G and Low-G signals for analysis and also to avoid alignment error or placement difference which could affect PTA measurements [16]. Furthermore, ±16 *g* is the most common operating range for accelerometers used in running gait analysis [10], and it would therefore be most relevant to validate the restoration algorithm within this operating range. In a published study that examined the effect of the accelerometer operating range on axial PTA, it was recommended that sensors utilize a minimum operating range of ±16 *g* [13]. However, results of the current study suggest that ±16 *g* may not be sufficient considering that we found a significant difference between the values obtained from the ±16 *g* and the ±200 *g* accelerometer. Specifically, the axial and resultant peaks were both found to be significantly smaller when using the Low-G sensor without the restoration algorithm, with small to large effects. We also observed larger effect sizes in participants with higher PTA values, which aligns with Ziebart et al.’s findings [31]. Additionally, while our results cannot be compared directly to Mitschke et al.’s study [13], we found similarity in that the lower operating range sensor results in significantly lower values. From the histogram of peaks for individual participants (Figure 1), we observed an inflated number of peaks around the 13–16 *g* range. When the acceleration exceeds the operating range, the Low-G accelerometer outputs the maximum value, in this case 16 *g*, forming a flat-cut-off. The “peak” was identified as the maximum value within the first 40% of a stride after the signal has been filtered, hence incorrectly registering “peaks” within the range 13–16 *g*. Based on this observation, we recommend researchers to inspect their data thoroughly for clipping, especially when the data are skewed toward the maximum operating range. Readers should also exercise caution when interpreting results of a study that used low operating range accelerometers.

The intention of this study is to validate the restoration algorithm which could provide a solution for researchers with PTA data collected using low operating range accelerometers. However, our findings found wide LOA and large mean difference between the reconstructed peaks. A high operating range accelerometer is recommended for dependable PTA measurements. Machine learning-based techniques, including convolutional neural network autoencoders trained with tri-axial acceleration data [32], could be investigated in future studies. It should be noted that the data sets of all participants were pooled together. We did not sub-divide the data set by surface inclination or footstrike pattern. Both are factors that could influence the magnitude and timing of the peaks in the x-axis (antero-posterior) and the resultant PTA [28,33]. Another limitation of the present study is the use of two accelerometers; while they were both encased within the same device, they had different resolutions. The resolution is lower for the High-G accelerometer, and there could be a difference in the PTA measurements between the accelerometers.

## 5. Conclusions

In conclusion, both the axial and resultant peaks derived from the restored signals were found to be smaller than the true value. The LOA for axial PTA was found to be larger with higher peak magnitudes. The use of the ±16 *g* accelerometer with or without the restoration algorithm for the measurement of axial or resultant PTA could result in invalid measurements and lead to incorrect conclusions. Our results are important for researchers in deciding the optimal accelerometer specification for their future investigation. The LOA reported in this study should be consulted when using accelerometers with low operating ranges with the restoration algorithm.

## Data Availability

The data presented in this study are openly available in FigShare at https://doi.org/10.6084/m9.figshare.22490872.v1 (accessed on 31 March 2023).

## Supplementary Materials

The following supporting information can be downloaded at: https://www.mdpi.com/article/10.3390/s23104609/s1, Supplementary File S1: Data processing flow diagram; Supplementary File S2: MATLAB script for signal restoration; Supplementary File S3: Histograms of axial peaks for all participants.
