# Supplementary material for: Evaluation of a Restoration Algorithm Applied to Clipped Tibial Acceleration Signals
Source: Sensors (Basel). 2023 May 10;23(10):4609. doi: 10.3390/s23104609 (PMC10222349; doi:10.3390/s23104609)

Supplementary File S1

Data processing flow diagram

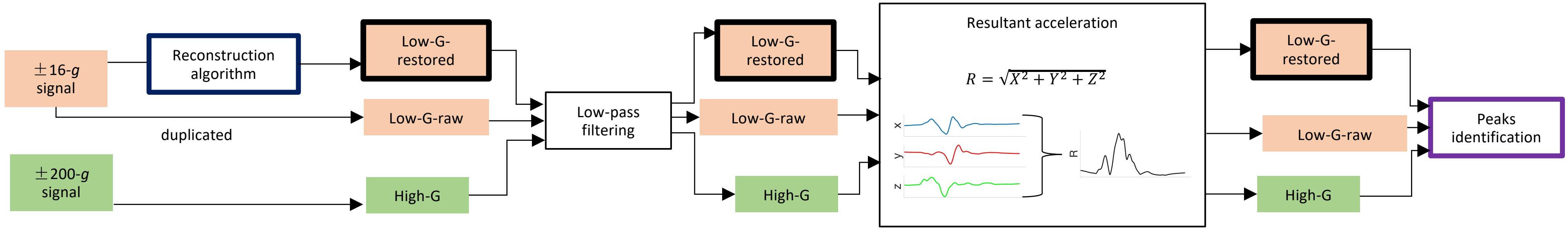

Reconstruction algorithm

At each axis (x, y and z),

1. The clipped portion is identified from raw signal (solid line),
2. Three data points before and after the clipped portion (as indicated by black dots) were used for interpolation, and
3. Signal at the clipped portion was reconstructed (dotted line) by 5th order spline interpolation

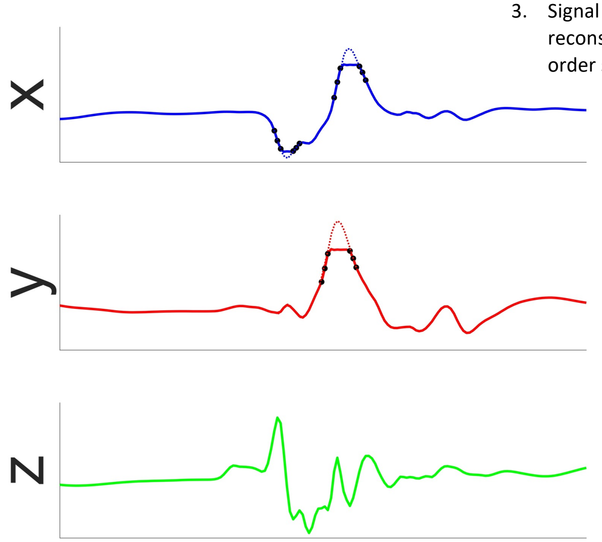

Peaks identification

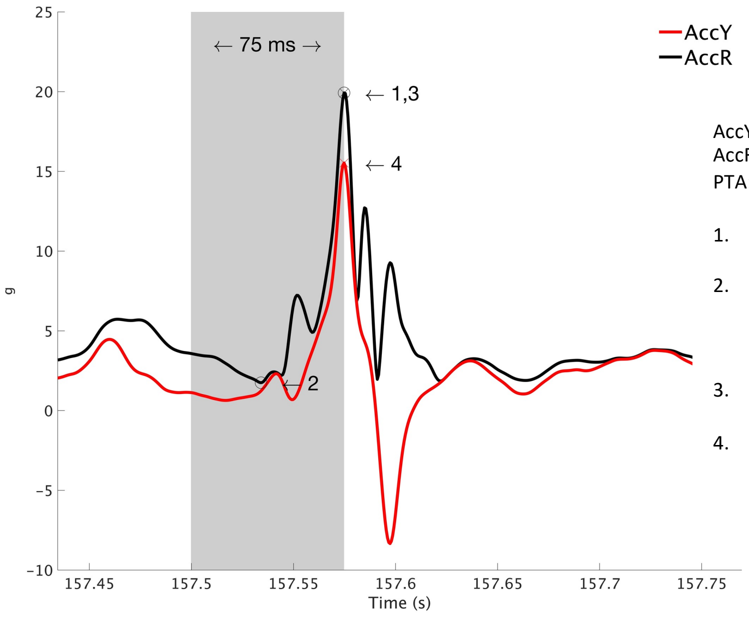

AccY: axial acceleration signal
AccR: resultant acceleration signal
PTA: peak tibial acceleration

1. Local maximum in resultant acceleration
2. Initial foot contact: local minimum of resultant acceleration that occurred within 75 ms of prior to point 1 (shaded region)
3. Resultant PTA: maximum value for resultant acceleration
4. Axial PTA: maximum value for axial acceleration

Supplementary File S2

MATLAB script used for restoration.

Sample data and a copy of the MATLAB script can be downloaded at FigShare:
https://doi.org/10.6084/m9.figshare.22720327

```
axis_n={'x','y','z'};

load('sample.mat')

tempLowG = sample.lowG_raw;
tempHighG = sample.highG_raw;

for axisn=1:3     %convert ms^-2 > g
    tempLowG.(['a' axis_n{axisn} '_g']) = tempLowG.(['a' axis_n{axisn}
'_mss'])/9.81;
    tempHighG.(['a' axis_n{axisn} '_g']) = tempHighG.(['a' axis_n{axisn}
'_mss'])/9.81;
end
sample.lowG_raw = tempLowG;
sample.highG_raw = tempHighG;

clipThreshold = 15.985; % value of flat cut-offs, to identify clipped regions

for axisn=1:3
    recon=[];
    clear temp2 temp3 tooHighTemp tooLowTemp outOfBounds inBound

    tooHighTemp=find(tempLowG.(['a' axis_n{axisn} '_g'])<=-clipThreshold); %
identify upper (~16-g) flat cut-offs
    tooLowTemp=find(tempLowG.(['a' axis_n{axisn} '_g'])>=clipThreshold); %
identify lower (~ 16-g) flat cut-offs

    outOfBounds=sort([tooHighTemp; tooLowTemp]);
    inBound=find(~ismember(1:size(tempLowG,1),outOfBounds))';

    %Uncomment below to plot points identified as clipped sections (red
    %crosses)
    %          plot(tempLowG.time_s,tempLowG.ay_g)
    %          hold on
    %
plot(tempLowG.time_s(outOfBounds),tempLowG.ay_g(outOfBounds),'rx')

    temp2=[tempLowG.time_s(inBound) tempLowG.(['a' axis_n{axisn}
'_g'])(inBound) inBound]; % signal to be preserved
    temp3=[tempLowG.time_s(outOfBounds) tempLowG.(['a' axis_n{axisn}
'_g'])(outOfBounds) outOfBounds]; % clipped portion

    temp4=[]; temp5=[]; upE=[]; downE=[];
    winSize=3; % number of points to use before and after the identified
clipped portion


    for n=1:size(temp3,1)
```

```
        % temp4 are edges, time_s acc_g frame (1:3 are up, and 4:6 are down)
        % temp5, 3 before and 3 after, layer 1: time, layer 2: Acc in g

        % identify beginning of clipped portion
        upE=find(temp2(:,3)<temp3(n,3),1,'last');
        if temp3(n,3)-1==temp2(upE,3)
            temp4(end+1,1:3)=temp3(n,:);
            temp5(size(temp4,1),1:winSize,1)=tempLowG.time_s(temp3(n,3)-
winSize:temp3(n,3)-1);
            temp5(size(temp4,1),1:winSize,2)=tempLowG.(['a' axis_n{axisn}
'_g'])(temp3(n,3)-winSize:temp3(n,3)-1);
        end
        % identify end of clipped portion
        downE=find(temp2(:,3)>temp3(n,3),1);
        if temp3(n,3)+1==temp2(downE,3)
            temp4(end,4:6)=temp3(n,:);

temp5(size(temp4,1),winSize+1:winSize*2,1)=tempLowG.time_s(temp3(n,3)+1:temp3
(n,3)+winSize);
            temp5(size(temp4,1),winSize+1:winSize*2,2)=tempLowG.(['a'
axis_n{axisn} '_g'])(temp3(n,3)+1:temp3(n,3)+winSize);
        end
    end

    temp2=temp2(upE:end);
    freq=1125; %lowg sampling frequeny

    for n=1:size(temp5,1)
        clear xx yy
        x=temp5(n,1:winSize*2,1); % time (raw)
        y=temp5(n,1:winSize*2,2); % acceleraion signal used for interpolation
(raw)
        xx=[temp5(n,1,1)
temp5(n,winSize,1)+1/freq:1/freq:temp5(n,winSize+1,1)-1/freq
temp5(n,winSize*2,1)]; % time (after interpolation)
        S = spapi(5,x,y); % 5th order Spline
        S = fnval(xx,S)'; % acceleration signal after interpolation

        periodT=[];

        % combining the preserved (unaltered) part of signal with
        % interpolated signal
        if n==1 % from start
            periodT=find(tempLowG.time_s<temp5(n,1,1));
            recon=[tempLowG.time_s(periodT) tempLowG.(['a' axis_n{axisn}
'_g'])(periodT); xx' S];
            %              if size(temp5,1)==1
            %                  periodTend=find(tempLowG.time_s>temp5(n,6,1));
            %                  recon=[recon; tempLowG.time_s(periodTend)
tempLowG.(['a' axis_n{axisn} '_g'])(periodTend)];
            %              end
        elseif n==size(temp5,1) % to end
            periodT=find(tempLowG.time_s>temp5(n-1,winSize*2,1) &
tempLowG.time_s<temp5(n,1,1));
            periodTend=find(tempLowG.time_s>temp5(n,winSize*2,1));
```

```
            recon=[recon; tempLowG.time_s(periodT) tempLowG.(['a'
axis_n{axisn} '_g'])(periodT); xx' S; tempLowG.time_s(periodTend)
tempLowG.(['a' axis_n{axisn} '_g'])(periodTend)];
        else % in between
            periodT=find(tempLowG.time_s>temp5(n-1,winSize*2,1) &
tempLowG.time_s<temp5(n,1,1));
            recon=[recon; tempLowG.time_s(periodT) tempLowG.(['a'
axis_n{axisn} '_g'])(periodT); xx' S];
        end


    end
    if isempty(recon) % if no clipped portion identified
        recon=[tempLowG.time_s tempLowG.(['a' axis_n{axisn} '_g'])];
    end
    sample.lowG_recon.(['a' axis_n{axisn}])=recon;

    subplot(3,1,axisn)
    plot(sample.lowG_raw.time_s,sample.lowG_raw.(['a' axis_n{axisn}
'_g']),':')
    hold on
    plot(sample.lowG_recon.(['a' axis_n{axisn}])(:,1),sample.lowG_recon.(['a'
axis_n{axisn}])(:,2))
    plot(sample.highG_raw.time_s,sample.highG_raw.(['a' axis_n{axisn} '_g']))
    box off
    if axisn == 1
        legend('LowG-raw','LowG-restored','HighG')
    end
end
```

Supplementary File S3

Histogram of axial peaks obtained from the Low-G-raw and Low-G-restored signal and the true value for all participants.

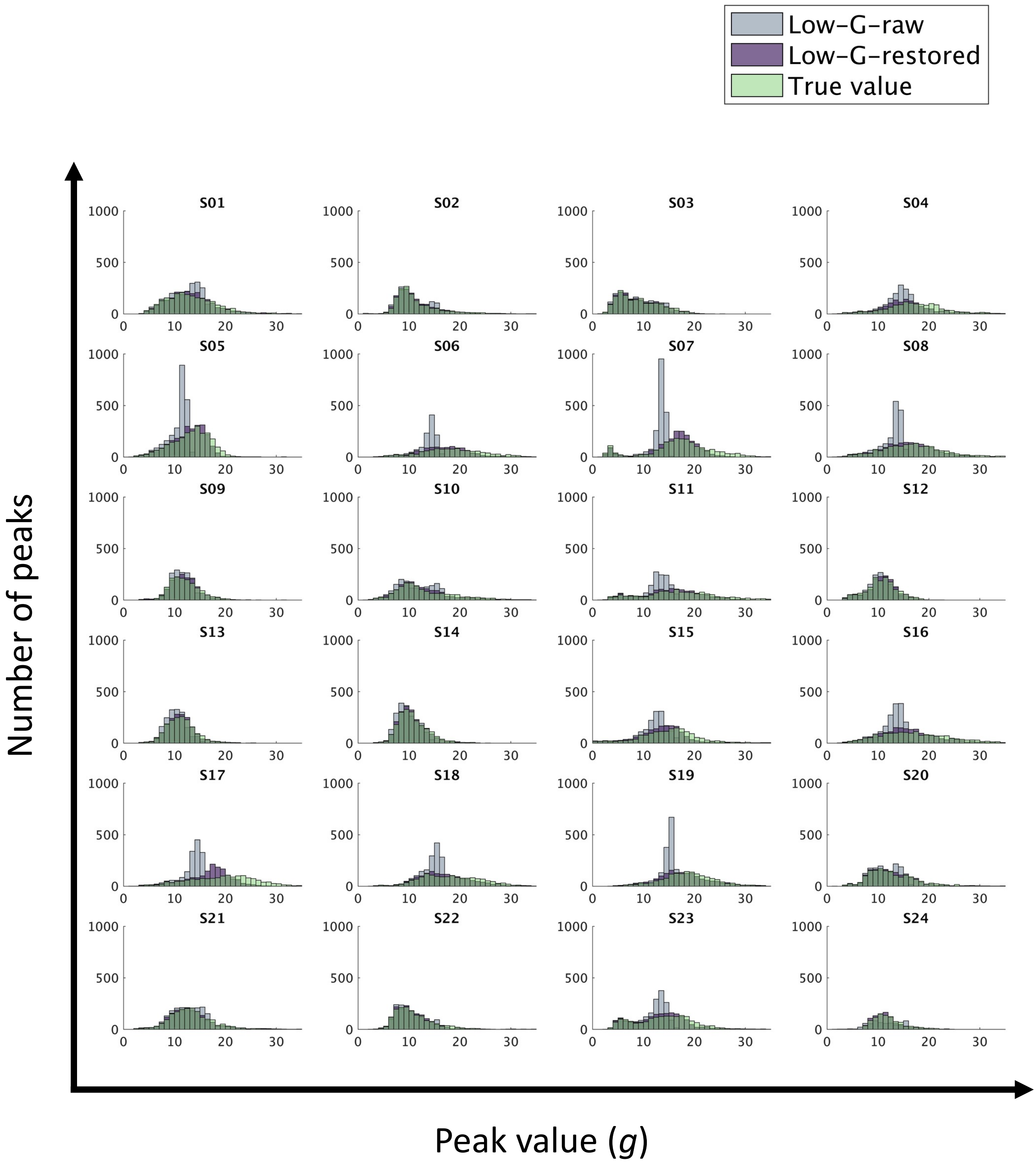

Supplement: Supplementary file 1 [file sensors-23-04609-s001.zip › sensors-2353021-supplementary.pdf]
